# Automated detection and classification of polioviruses from nanopore sequencing reads using piranha

**DOI:** 10.1093/ve/veae023

**Published:** 2024-02-29

**Authors:** Áine O’Toole, Rachel Colquhoun, Corey Ansley, Catherine Troman, Daniel Maloney, Zoe Vance, Joyce Akello, Erika Bujaki, Manasi Majumdar, Adnan Khurshid, Yasir Arshad, Muhammad Masroor Alam, Javier Martin, Alexander G Shaw, Nicholas C Grassly, Andrew Rambaut

**Affiliations:** Institute of Ecology & Evolution, University of Edinburgh, Edinburgh EH93FL, UK; Institute of Ecology & Evolution, University of Edinburgh, Edinburgh EH93FL, UK; Institute of Ecology & Evolution, University of Edinburgh, Edinburgh EH93FL, UK; Department of Infectious Disease Epidemiology, Imperial College London, London W120BZ, UK; Institute of Ecology & Evolution, University of Edinburgh, Edinburgh EH93FL, UK; Institute of Ecology & Evolution, University of Edinburgh, Edinburgh EH93FL, UK; Department of Infectious Disease Epidemiology, Imperial College London, London W120BZ, UK; Division of Virology, National Institute for Biological Standards and Control (NIBSC), Potters Bar EN63QG, UK; Division of Virology, National Institute for Biological Standards and Control (NIBSC), Potters Bar EN63QG, UK; Department of Virology, National Institute for Health, Islamabad 44000, Pakistan; Department of Virology, National Institute for Health, Islamabad 44000, Pakistan; Department of Virology, National Institute for Health, Islamabad 44000, Pakistan; Division of Virology, National Institute for Biological Standards and Control (NIBSC), Potters Bar EN63QG, UK; Department of Infectious Disease Epidemiology, Imperial College London, London W120BZ, UK; Department of Infectious Disease Epidemiology, Imperial College London, London W120BZ, UK; Institute of Ecology & Evolution, University of Edinburgh, Edinburgh EH93FL, UK

**Keywords:** poliovirus, surveillance, software, nanopore, VDPV, sabin, emerging disease, outbreak

## Abstract

Widespread surveillance, rapid detection, and appropriate intervention will be critical for successful eradication of poliovirus. Using deployable next-generation sequencing (NGS) approaches, such as Oxford Nanopore Technologies’ MinION, the time from sample to result can be significantly reduced compared to cell culture and Sanger sequencing. We developed piranha (poliovirus investigation resource automating nanopore haplotype analysis), a ‘sequencing reads-to-report’ solution to aid routine poliovirus testing of both stool and environmental samples and alleviate the bioinformatic bottleneck that often exists for laboratories adopting novel NGS approaches. Piranha can be used for efficient intratypic differentiation of poliovirus serotypes, for classification of Sabin-like polioviruses, and for detection of wild-type and vaccine-derived polioviruses. It produces interactive, distributable reports, as well as summary comma-separated values files and consensus poliovirus FASTA sequences. Piranha optionally provides phylogenetic analysis, with the ability to incorporate a local database, processing from raw sequencing reads to an interactive, annotated phylogeny in a single step. The reports describe each nanopore sequencing run with interpretable plots, enabling researchers to easily detect the presence of poliovirus in samples and quickly disseminate their results. Poliovirus eradication efforts are hindered by the lack of real-time detection and reporting, and piranha can be used to complement direct detection sequencing approaches.

## Introduction

**Figure 1.**
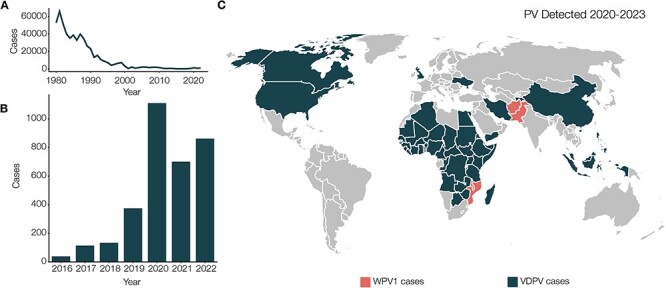
(A) Decline in poliovirus cases since 1980. The eradication initiative has helped reduce the incidence of poliovirus by 98 per cent since the 1980s. (B) In recent years, there has been an uptick in cases of poliovirus and re-emergence of the virus continues to be a concern. (C) Countries in which poliovirus has been detected between 2020 and 2023. WPV1 and circulating vaccine derived poliovirus (cVDPV) cases are indicated. VDPV cases include poliovirus type-1, type-2, and type-3 (VDPV1, VDPV2, and VDPV3). Data accessed from WHO.int and polioeradication.org on 2024–01-17. Incidence data from immunizationdata.who.int/pages/incidence/polio.html and polioeradication.org/thisweek/variant-polio-cvdpv-cases.

Following the success of smallpox eradication, great strides have been made towards poliovirus eradication. Vaccine rollout has contributed to a 99 per cent decrease in cases of poliovirus since 1988 (WHO.int accessed 22 November 2022; Fig. 1A); however, this eradication effort has been hindered by re-emergence of vaccine-derived poliovirus (VDPV). Recent trends indicate an uptick in cases of poliovirus since 2016 (Fig. 1B), and since 2020, VDPV has been detected in Africa, Asia, North America, and Europe (Fig. 1C). There are excellent poliovirus surveillance networks across the world, with the Global Polio Laboratory Network (GPLN) represented in ninety-two countries. For detection of nascent outbreaks, it is important to determine the type of poliovirus circulating in order to implement appropriate intervention.

Standard laboratory algorithms for this characterisation involve culturing stool or environmental samples in poliovirus-susceptible cell lines, intratypic differentiation (ITD) by quantitative polymerase chain reaction, and then Sanger sequencing of the programmatically important isolates (WHO 2017; Shaw et al. 2022). This approach is regarded as the gold standard; however, on average, it can take 2–3 weeks for cell culturing results. Factoring in transit to a specialised sequencing facility, the median detection time in countries across Asia and Africa ranges from 3 to 6 weeks (WHO. Global Polio Eradication Initiative 2020). This lag between sample collection and reporting involved with traditional detection approaches has hindered eradication efforts. As such, a major objective of the Global Polio Eradication Initiative is to adopt a robust and sensitive, culture-independent ‘direct detection’ approach for poliovirus detection and ITD. A number of approaches have been trialled, including a capture assay with recombinant His-tagged protein and an approach involving direct RNA extraction and reverse transcription (rt)-PCR (Gerloff et al. 2021; Harrington et al. 2021). Shaw et al. proposed an alternative detection method that bypasses the need for cell culture and enables direct detection of poliovirus from samples using Nanopore sequencing (Jain et al. 2016; Shaw et al. 2020). Nanopore is a long-read technology, which enables us to sequence the VP1 region of poliovirus genomes in a single amplicon (Shaw et al. 2020). Using this direct detection and nanopore sequencing (DDNS) method to perform sequencing of poliovirus locally within the country of sample testing could result in significant decreases in the time required to detect programmatically important viruses, providing the opportunity for more rapid outbreak response and earlier truncation of transmission (Shaw et al. 2022).

We are in an era of real-time genomic surveillance. The coronavirus disease (COVID)-19 pandemic illustrated that real-time genomic surveillance is possible and set a precedent for open data sharing practices too. Next-generation sequencing capacity across the globe has grown enormously in the last few years. While bioinformatic capacity has also improved, it is still often a major hurdle to implementing next-generation sequencing in house. The difficulty lies in maintaining a robust and consistent analysis set-up, particularly across the entire GPLN where fine-scale analysis is critical to determining whether an outbreak is caused by a VDPV or wild-type poliovirus (WPV) and what should be the appropriate response.

With efficient detection and reporting of poliovirus, governments and health agencies can make informed decisions regarding outbreak control within a timeframe that may help change the course of a poliovirus outbreak, truncating Sabin-related transmission chains before they have an opportunity to re-emerge as pathogenic poliovirus. We present piranha (poliovirus investigation resource automating nanopore haplotype analysis), a novel software tool that provides the means for maintaining consistent, replicable, validated analysis of poliovirus sequencing data for both stool sample detection and environmental surveillance.

## Methods

Piranha is a tool developed as part of the Poliovirus Sequencing Consortium (polio-nanopore.org) to help standardise and streamline analysis of nanopore-based poliovirus sequencing, particularly DDNS. It is Python-based with an embedded analysis pipeline built in a Snakemake framework (Köster and Rahmann 2012). The output of piranha includes an interactive report summarising the entire Nanopore sequencing run, a detailed report for each sample within a sequencing run, the output VP1 sequences detected within each sample, variant call support values, and phylogenetic trees produced using the software. Piranha by default is configured to analyse nanopore VP1 sequences generated from the sequencing protocol described in Shaw et al. (2020). However, it can be used to analyse whole-genome sequences too. It can produce reports in either English (default) or French to aid uptake by laboratories across the GPLN. Documentation describing installation, full usage, configuration options, and some example data for piranha is available at https://github.com/polio-nanopore/piranha.

### Deployment

Piranha has been developed with ease of deployment in mind and with the knowledge that availability of a skilled bioinformatician will be heterogeneous across the GPLN and in other public health laboratories settings. Piranha is a flexible command line tool that can have most default settings and reference files modified through either command line flags or a configuration file. This flexibility may be desired by laboratories trialling novel detection approaches and would require an individual with experience of the command line. However, for use in the GPLN, piranha can be easily run without use of the command line through piranhaGUI (github.com/polio-nanopore/piranhaGUI), our custom-designed user interface for piranha that handles installation and operating system compatibility problems It has also been configured to run as part of Epi2Me (labs.epi2me.io), Nanopore’s user-friendly user interface (UI), and can be imported and run there as a workflow.

A high-performance laptop with a graphics processing unit (GPU) is necessary for running MinION sequencing. We recommend running piranha on the same machine used for sequencing; however, a GPU is not necessary for running piranha. Sequencing read data produced will need to be stored for the GPLN recommended period of time—currently 2 years—and as such, sufficient storage capacity (∼10 TB) is recommended. These one-off costs are detailed in the protocols.io workspace (https://www.protocols.io/workspaces/poliovirus-sequencing-consortium).

Installation of piranhaGUI is very straightforward and can be run on Windows and MacOS machines. Docker is the only external dependency required for piranhaGUI (https://docs.docker.com/get-docker/), and this can also be installed without the use of the command line. Piranha is intentionally less configurable through piranhaGUI to ensure that the workflow is run with recommended settings across the GPLN. Guidance on installation and use of piranha through piranhaGUI can be found on protocols.io at dx.doi.org/10.17504/protocols.io.ewov1q642gr2/v1.

Skills required by the user of piranha include a basic understanding of genomics, as well as proficiency in using the software interface and interpreting its results. Any laboratory member who can use interface-based software, such as the Oxford Nanopore Technologies (ONT) MinKNOW interface, will be able to use piranhaGUI. We provide support, any general issues or technical queries that arise regarding the adoption of DDNS can be flagged with the consortium via the protocols.io workspace (protocols.io/workspaces/poliovirus-sequencing-consortium), and issues regarding piranha software can be directly flagged on the repository at github.com/polio-nanopore/piranha.

### Input files

**Figure 2.**
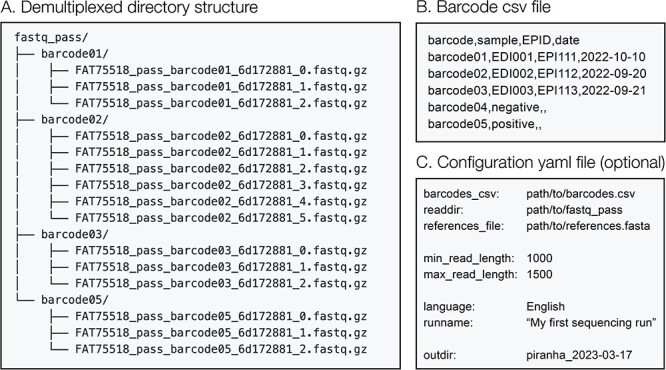
Piranha input files and format. (A) Piranha detects fastq (or fastq.gz) files within the demultiplexed read directory specified, in this example, ‘fastq_pass’. Within that directory, piranha looks for subdirectories with each barcode name and processes all read files within them. Specify the input read directory with -i/--readdir. (B) The user must supply a CSV file containing minimally a column describing which barcode and a column mapping a barcode onto a sample ID (-b/--barcodes-csv). Additional metadata can also be supplied, such as in this example EPID or date. (C) Users can optionally supply a configuration file (-c/--config) with all command line parameters specified.

Piranha processes nanopore sequencing read files in FASTQ format. Current gold standard for nanopore sequencing analysis is to first basecall and demultiplex raw sequence data using Guppy within MinKNOW (Wick, Judd, and Holt 2019). As such, piranha is configured to detect and access FASTQ read data within the specified directory in the format that Guppy produces (subdirectories that correspond to each barcode in the sequencing run with fastq or fastq.gz sequencing read files; Fig. 2A). A barcode comma-separated values (CSV) file (Fig. 2B) must be supplied, which informs piranha which barcodes to analyse. Minimally, this file must contain information matching a barcode ID (in the same format as the read directory output by Guppy) to a sample ID. Additional metadata can be supplied in this file, and it will be incorporated into the final output of piranha to aid in the interpretation of results and data management. For example, in Fig. 2B, EPID (a unique case identifier) and date have been included as optional metadata columns. There are many configuration options available in piranha, with default settings tailored to the DDNS protocol. All options can be configured with command line flags or can be supplied in a configuration file (Fig. 2C).

### Background database

**Figure 3.**
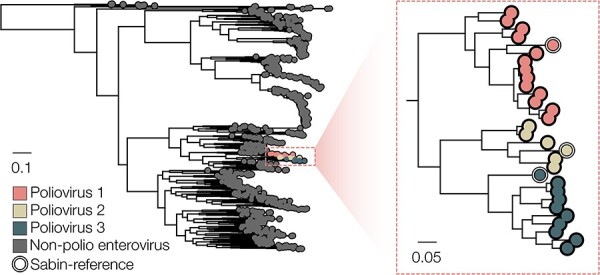
VP1 reference database composition represented as a phylogeny (distance bar indicates nucleotide substitutions per site). To construct the phylogeny, we aligned the reference sequences using MAFFT (Katoh and Standley 2013), visually inspected the resulting alignment, and used iqtree2 to estimate a maximum-likelihood phylogeny with a Hasegawa-Kishino-Yano (HKY) substitution model, representing the diversity within the background database (Minh et al. 2020). The background database consists of a range of human-infectious non-polio enterovirus sequences, representatives of wild-type poliovirus serotypes 1, 2, and 3 and the vaccine reference strains Sabin 1, Sabin 2, and Sabin 3 (*n* = 959).

Within the analysis framework of piranha, noisy nanopore sequencing reads are matched against a reference database using minimap2 (with map-ont mode and no secondary chains reported) (Li 2018). The default reference database in piranha contains a representative set of 959 publicly available sequences for poliovirus type 1, 2, and 3 and non-polio enterovirus VP1 sequences collected from National Center for Biotechnology Information and Virus Pathogen Resource, now part of bv-brc.org (Pickett et al. 2012) (Fig. 3; https://github.com/polio-nanopore/piranha/blob/main/piranha/data/references.vp1.fasta). The reference sequences come installed as part of the piranha software, but can also be accessed at github.com/polio-nanopore/piranha. All three Sabin vaccine reference sequences are included in the default database.

### Analysis pipeline

**Figure 4.**
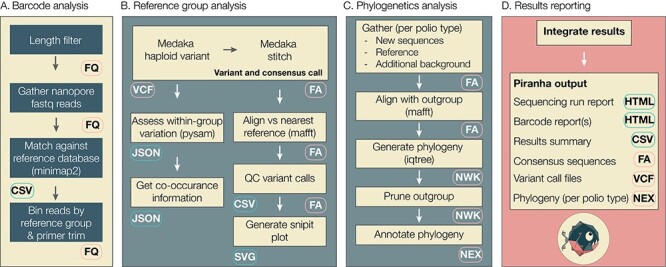
Piranha workflow schema. (A) Initial analysis is done for each barcode specified in a barcodes CSV file. Reads are mapped against a background reference FASTA file and categorised into broad ‘reference group’ categories: wild-poliovirus 1 (WPV1), WPV2, WPV3, Sabin 1–like polioviruses, Sabin 2–like polioviruses, Sabin 3–like polioviruses, or NonPolioEV. (B) For each reference group detected within a sample, variants are called against the relevant references using medaka and consensus sequences are generated. Within-group variation and co-occurrence information are estimated, and quality control was performed on the variant calls. Snipit plots are also generated based on reference-consensus alignments for each reference group. (C) Piranha has an optional phylogenetics module that will gather a set of sequences for every ‘reference group’ category that has been detected in the sequencing run (including all samples). Each set contains the newly generated consensus sequences of a given reference group, the relevant reference itself, and any additional background sequences that have been provided. The sequences are aligned with an appropriate outgroup, and a maximum-likelihood phylogeny is generated. The outgroups are pruned off, and the phylogeny is annotated with configurable metadata fields. (D) Piranha outputs an interactive summary report and accompanying summary CSV, and it also produces detailed interactive reports for each barcode and the consensus sequences and variant call files for each reference group detected within a barcode. It also provides each phylogeny file, alongside displaying them in the interactive report. File type abbreviations: FQ (FASTQ), FA (FASTA), VCF (variant call files), NWK (Newick), and NEX (Nexus).

Piranha first reads in the barcode.csv file checks for unique barcode and sample names and compiles a set of barcodes to analyse. It checks the read path and ensures that there are demultiplexed FASTQ files found that correspond to the barcodes it has been instructed to analyse. For each noisy nanopore sequencing read (FASTQs) file, piranha filters by read length to remove any chimeric or off-target reads (Fig. 4A). The default length filter is tailored to the VP1 protocol and filters out reads less than 1,000 or greater than 1,500 nucleotide bases long. These defaults can be overwritten by customising the read lengths (--min-read-length, --max-read-length) or by using the --analysis-mode flag (qnalysis modes for pan-enterovirus (panev) filters read lengths outside the range of 3000–4500 nucleotide bases and whole-genome mode (wg) filters for lengths 3400–5200). The length-filtered reads are written to an aggregated read file, one for each barcode specified. This aggregated file is only created transiently unless piranha is run in no-temp mode (specified with --no-temp).

Within piranha, each aggregated FASTQ read file is mapped against a FASTA database of reference sequences described in Fig. 3. Alternative reference sequences in FASTA format can be supplied to piranha (-r flag). Within piranha, these diverse references are categorised into the following: ‘ddns_group’ bins for further analysis: Sabin 1–like, Sabin 2–like, and Sabin 3–like polioviruses, WPV1, WPV2, and WPV3 and non-polio enterovirus (NonPolioEV) (Fig. 4A). This grouping can be configured by the user of command line piranha if individual enterovirus species are of interest too by supplying the reference group argument (-rg). Mapped reads must be within the minimum and maximum read length and have a mapping quality score greater than the minimum mapping quality (default *Q* = 50).

For each barcode, any reference group category that has more than a minimum number of reads and a minimum percentage of sample is taken forward to reference group analysis (Fig. 4B). For each set of reads, piranha first trims the primer sequences (primer length configurable) and then uses ONT’s medaka software to variant and consensus call the noisy nanopore reads against the relevant reference sequence (Oxford Nanopore Technologies 2019). For Sabin-related reads, they are variant-called against the respective Sabin reference sequence (1, 2, or 3). For wild-type polio and non-polio enterovirus sequences, the reference with the highest number of reads mapping for a given category is used for the variant calling step, resulting in the majority consensus sequence present in a sample for each reference group. Within-group variation is calculated using pysam (https://github.com/pysam-developers/pysam), and we calculate a co-occurrence matrix for variants above the minimum percentage threshold. These thresholds are all configurable parameters either by the command line or through the yaml file. A reference sequence vs consensus sequence is constructed using MAFFT (Katoh and Standley 2013), and biologically implausible variants are replaced with N as they may be a result of sequencing error rather than genuine mutations (e.g. frameshift insertion or deletion mutations in the VP1 coding sequence). Piranha generates snipit plots (https://github.com/aineniamh/snipit) based on the alignments to highlight the differences between reference and sample sequences. This is particularly important for categorising Sabin-related sequences into Sabin polioviruses, Sabin-like polioviruses, or VDPV. For Sabin 1–related and Sabin 3–related sequences, VP1 sequences with ten or more mutations relative to the Sabin reference are classified VDPV sequences, whereas for Sabin 2–related sequences, the threshold is 6 or more mutations (WHO 2016).

An optional phylogenetics module has been implemented for piranha (initiated by using the -rp/--run-phylogenetics flag) that generates maximum-likelihood phylogenies for each ‘reference group’ detected within a sequencing run (i.e. Sabin 1–related polioviruses, Sabin 2–related polioviruses, Sabin 3–related polioviruses, WPV1, WPV2, or WPV3). This module allows the user to easily compare samples within the same sequencing run and also to integrate additional sequences from a local database (Fig. 4C). To supply additional sequences and (optionally) an accompanying metadata file, the user can use the -sd/--supplementary-data flag and provide a directory with additional sequences and metadata. Piranha first gathers a set of sequences, including all new consensus sequences for a given reference group generated during the run, references present in the reference file within piranha, and any additional supplementary sequences of a given reference type that the user supplies. Piranha then aligns these against an outgroup sequence (the relevant Sabin reference sequence for a given poliovirus type) using MAFFT and estimates a phylogeny using iqtree2 (Katoh and Standley 2013; Minh et al. 2020). The outgroup is pruned off and each phylogeny annotated with metadata that can be configured with the -pcol/--phylo-metadata-columns flag. These phylogenies are displayed in the interactive report produced by piranha and are also supplied as nexus (NEX) files.

**Figure 5.**
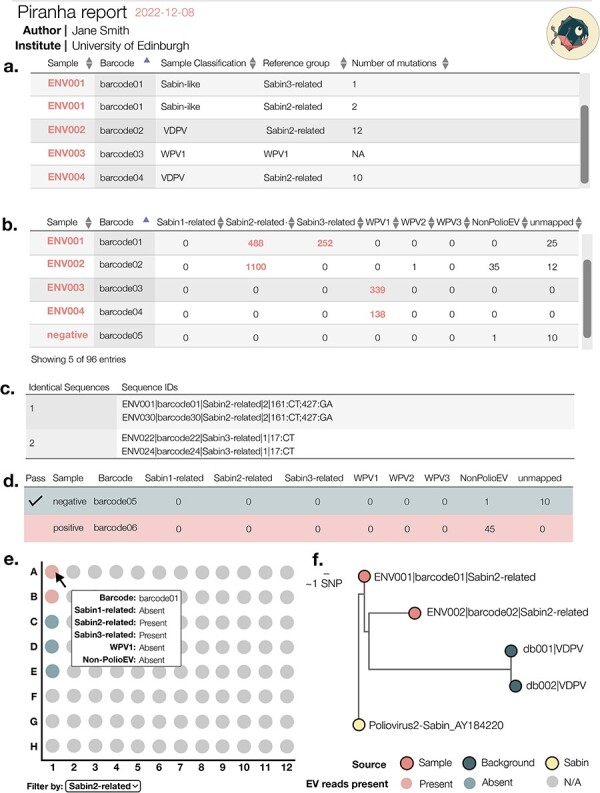
Schema of a piranha output summary report. The report is an interactive, distributable file and can be personalised, with the ability to add a custom report title, username, and institute to the header of the report. (A) The first table in the report gives an overview of which enteroviruses were detected in which samples in the sequencing run. For Sabin-related polioviruses, the classification and number of mutations from Sabin are reported. (B) The second table includes a summary of the read counts mapped against each reference group for each barcode. (C) Any identical sequences present in multiple samples are flagged for investigation as possible contaminants across samples. (D) Samples specified as negative or positive controls in the barcodes.csv file are flagged with a tick if the control passes. For the negative control, samples must have not more than the minimum read threshold mapped in any reference group. For the positive control to pass, samples must have more than the minimum read threshold mapped to the NonPolioEV category since the positive control consists of Coxsackievirus A-20, which is amplified by the DDNS nested polymerase chain reaction (PCR). (E) The protocol by Shaw et al. (2020) utilises a 96-well plate set-up (Shaw et al. 2020). The piranha report visualises this plate, with barcodes arranged in a configurable horizontal or vertical set-up. The presence and absence of different reference groups is indicated to highlight clustering on the plate, again in an effort to flag potential contamination across samples. (F) Each phylogeny produced is displayed in the report as an interactive tree that can be expanded and coloured by various metadata fields and has information about each tip in the phylogeny that can be brought up by clicking the tip.

Piranha produces a number of final results (Fig. 4C), by default intermediate workings created in a temporary directory that gets deleted upon completion, but all intermediate files can be retained with the—no-temp flag. Piranha outputs the consensus fasta files and variant call files for each reference group present in each barcode, an overall summary CSV for the entire run, an interactive report summarising and visualising the entire sequencing run (Fig. 5), and a detailed

**Figure 6.**
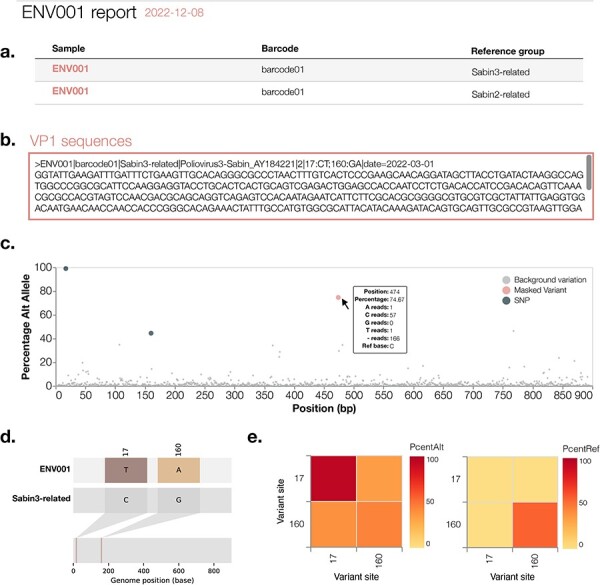
Schema of the detailed piranha report produced for each sample in a sequencing run (here, sample ENV001). (A) The first table summarises all reference groups detected in that sample. (B) A scrollable box with the consensus FASTA sequences produced for this sample. The sequence record header content is configurable within piranha. (C) For each reference group detected, piranha produces a within-group variation plot that shows the percentage of sequencing reads with an allele distinct from the reference allele. The figure highlights the mutations that ONT’s medaka called in a given consensus FASTA sequence and any mutations that may have been masked out by piranha (e.g. a frameshift mutation in VP1). The background % alternative allele variation is displayed as the smaller grey points. This plot highlights whether there is potential for a mixed sample, as in this example, the SNP at Position 17 is at 100 per cent frequency; however, the SNP called by medaka at Position 160 is at ∼40 per cent frequency. (D) Piranha produces and displays snipit plots, which summarise differences with respect to each reference group detected within a sample. (E) Co-occurrence plots summarise the percentage alternative allele and percentage reference for each of the variants called by ONT’s medaka.

Report for each barcode (Fig. 6). Example reports are hosted at http://polionanopore.org/piranha/report.html.

## Results

### Piranha can reliably detect poliovirus1, poliovirus2, and poliovirus3 from stool and accurately catalogue the count of SNPs from Sabin for classification of VDPVs

**Figure 7.**
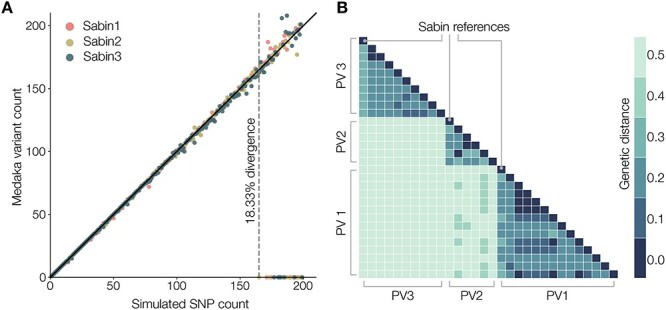
(A) We simulated increasing numbers of mutations in a Sabin 3 reference away from the actual virus sequence. Variants were called using the medaka haploid variant model with 250 sequencing reads. At 18.33 per cent divergence (165 SNPs from the reference sequence), the reads begin to fail to map using minimap2. (B) Pairwise genetic distances (nucleotide substitutions per site) calculated from an alignment of representative Poliovirus (PV) 1, 2, and 3 VP1 sequences. Genetic distance within each Poliovirus group is lower than the observed mapping limit of minimap2.

We ran the piranha analysis pipeline on three sets of 200 real nanopore reads generated from sequencing laboratory strains of Sabin 1, 2, and 3, respectively, against modified reference sequences derived from Sabin sequences that contain simulated single nucleotide polymorphisms (SNPs). The core of the analysis pipeline is medaka haploid variant, which we found to be highly accurate in calling variants in a pure population of reads. We see little to no decline in variant calling ability as simulated SNP count increases for these pure samples until the limit of mapping is reached (Fig. 7). At 18.33 per cent divergence, we see minimap2 begin to fail to map the reads to the simulated reference. It is then as alignment accuracy has declined or failed completely that we see variant calls begin to be unreliable. From this finding, we can say that within the limits of minimap2’s ability to map reads reliably to a reference medaka should reliably call variants.

### Medaka haploid variant found to be the most suitable method for variant calling

**Figure 8.**
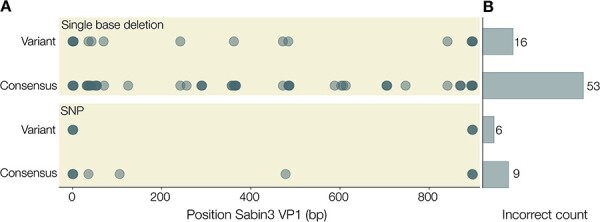
(A) We simulated references with either a single base deletion (top panel) or single-nucleotide polymorphism (SNP; bottom panel) different from the actual VP1 sequence a set of 250 amplicon reads represent. Points represent incorrect variant calls by either the medaka haploid variant or medaka consensus models of medaka. (B) Total count of incorrect variant calls for a given medaka model and type of mutation (deletion or SNP).

Within piranha, we wanted to use the most robust variant calling module possible while also considering long-term maintenance. Medaka is a tool developed by ONT that uses neural networks trained on nanopore data to call variants in a sample. Including medaka in piranha’s analysis pipeline was a deliberate choice. Nanopore technology is still in active development, and with chemistry and basecalling algorithms changing so frequently, it is important to implement the latest gold-standard analysis suite available for nanopore sequencing. With medaka, updates to the chemistry and upstream analysis algorithms can be co-opted in with the latest trained model. We assessed within medaka, which was the most appropriate algorithm for our purposes and found the medaka haploid variant module to be the best at calling SNPs and indels, although it was better at calling SNPs than indels (Fig. 8).

### No clear relationship between low-complexity regions and variant quality score

**Figure 9.**
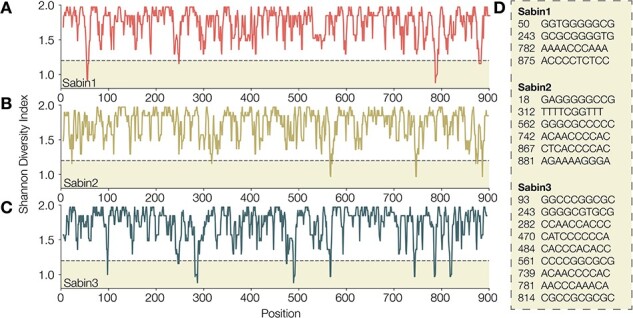
Low-complexity regions are highlighted by calculating Shannon Diversity Index in a 10-bp sliding window across the VP1 genes of (A) Sabin 1, (B) Sabin 2, and (C) Sabin 3. Diversity index scores less than 1.2 are highlighted, and the VP1 nucleotide position and nucleotides within the 10-bp window are indicated in (D).

**Figure 10.**
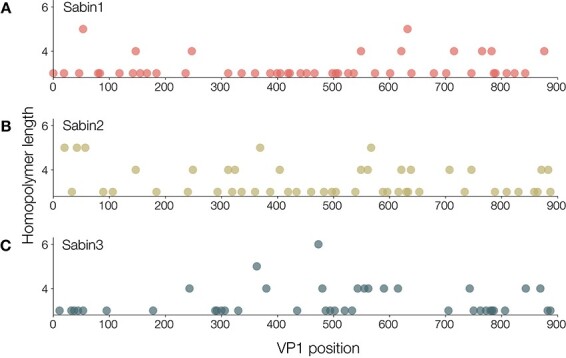
Length of homopolymers across (A) Sabin 1, (B) Sabin 2, and (C) Sabin 3. Nanopore technology struggles with homopolymeric runs. Here, we assess the length of homopolymers across the VP1 region for each Sabin reference in turn, to identify regions that may be problematic for variant calling. Homopolymers of length 3 or greater are displayed.

**Figure 11.**
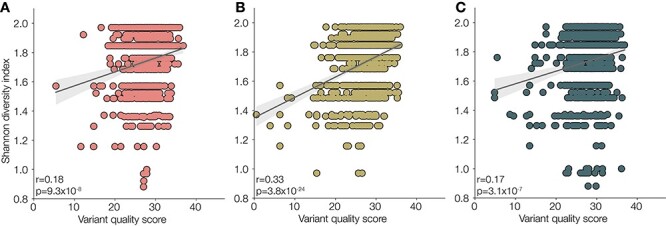
Shannon diversity of nucleotide context index plotted against the quality of the variant call for (A) Sabin 1, (B) Sabin 2, or (C) Sabin 3. Pearson’s *r* of the correlation and *P*-value are shown for each plot. The relationship is noisy, and variants with particularly low Shannon indexes are not necessarily the lowest quality variant calls.

We investigated whether low-complexity regions were more likely to give a lower variant quality score and whether variants called at these sites should be reported with the caveat of being within a low-complexity region. We first identified low-complexity regions by calculating the Shannon Diversity Index in a 10-bp sliding window across the VP1 region. In all three Sabin reference sequences (1, 2, and 3), we identified a number of low-complexity regions with a score of <1.2 (Fig. 9A–C). We confirmed that this index accurately captured low-complexity regions, with regions <1.2 shown in Fig. 9D, and confirmed that these regions captured most homopolymeric regions in the Sabin references (Fig. 10). Although there is a correlation between regions of low complexity and variant quality score, the relationship is noisy and likely could not be used to predict the quality of a variant (Fig. 11). As such, we provide the variant call files from ONT’s medaka with individual quality scores available for each variant called.

### Distinguishing between closely related references

**Figure 12.**
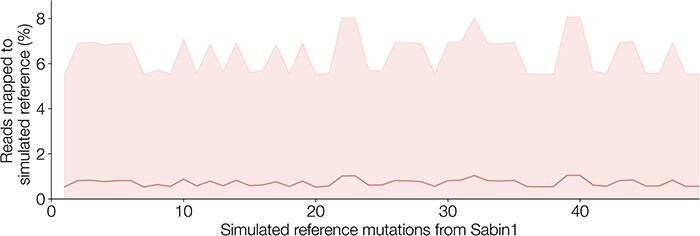
The proportion of Sabin 1 reads mapped to a synthetic (incorrect) reference plotted against the number of mutations separating that reference from the correct Sabin 1 reference. Minimap2 distinguishes between closely related references with very high accuracy. A set of 200 Sabin 1 sequencing reads were mapped (-x map-ont) against reference databases containing only a Sabin 1 reference and a simulated reference with X number of mutations from Sabin 1. For a given number of mutations, we simulated 200 randomly mutated references and so constructed 200 reference files and repeated up to fifty mutations from Sabin 1 (*n* = 10,000). The shaded regions represent standard deviation from the mean values for a given number of mutations, X.

We assessed how well we can distinguish between two closely related populations in piranha, which uses minimap2 to map against the reference database. We constructed reference databases of size two with a Sabin 1 reference strain and a simulated reference with a fixed number of mutations, X, added to the Sabin 1 reference. We simulated these reference databases for X in the range of 1 to 50 (i.e. a series of databases ranging from one containing Sabin 1 and a synthetic reference 1 mutation away from Sabin 1 and a database containing Sabin 1 and a synthetic reference fifty mutations away from Sabin 1). For each X, we randomly selected sites to mutate and repeated this 200 times, giving 10,000 test databases each containing two references (Sabin 1 and the synthetic reference). We tested how well minimap2 can map the Sabin 1 reads to the correct (Sabin) reference compared with the synthetic reference. We find that minimap2 assigns the correct reference with high fidelity (Fig. 12). Even at *X* = 1, when the reference sequences are only separated by a single SNP, <1 per cent of reads are assigned the (incorrect) synthetic reference and this remains consistent as the references become more distinct from one another.

### Distinguishing mixtures that have been created in silico

**Figure 13.**
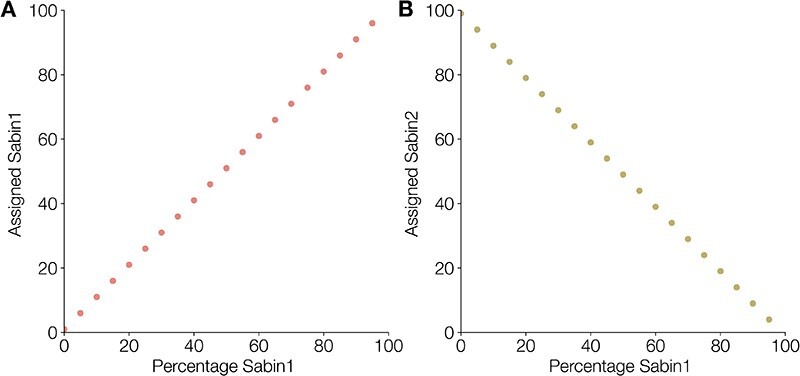
Minimap2 can perfectly resolve mixtures of Sabin 1 and Sabin 2 sequencing reads constructed *in silico*. As the percentage of Sabin 1 in the sample increases, the number assigned increases (A) and similarly the number of reads assigned Sabin 2 declines (B).

As a proof of principle, we also confirmed that minimap2 can distinguish between two distinct populations of reads representing different reference groups (in this case Sabin 1 and Sabin 2). Mixtures of real reads were constructed *in silico* at various proportions and minimap2 can resolve these mixtures accurately (Fig. 13).

## Discussion

Despite great strides towards polio eradication, nascent outbreaks arise and continue to persist in many parts of the world. Real-time surveillance and rapid outbreak response will be critical to the elimination of the disease. Deployable technologies for next-generation sequencing, such as the ONT MinION, can aid in rapid outbreak detection and inform interventions weeks earlier than the traditional cell culture approach (Shaw et al. 2022). However, these novel methods come with analytical challenges that may be a roadblock for laboratories considering adopting them. With piranha, researchers have access to best-practice, replicable bioinformatic processing and informative reports, making Nanopore sequencing for polio surveillance accessible to laboratories that do not necessarily have bioinformatics expertise.

Development of piranha focused primarily on detection and ITD of poliovirus from stool samples, with emphasis on classifying and identifying circulating VDPV. Piranha is very configurable, and although default settings are for VP1 sequencing, it can be used for constructing larger portions of the genome and whole-genome sequences. The current analytic default allows piranha to construct a single consensus sequence for each poliovirus serotype or Sabin-related poliovirus detected within a sample, but does have multiple figures illustrating the homotypic variation and co-occurrence frequency, enabling the user to interpret whether homotypic subpopulations exist within the data. We have implemented an experimental module for haplotype calling within serotype in piranha, and ongoing development includes benchmarking and validating this analysis to focus on finer-grain mixture resolution within serotypes in an effort to provide further resolution of homotypic mixtures in samples. We anticipate the rollout of Oxford Nanopore Technology’s R10.4.1 flow cells, and chemistry will aid these efforts significantly by reducing the noisiness of sequencing reads. For enterovirus-wide analysis, the main limitation of our current approach lies in the completeness of the default reference set that comes pre-installed with piranha. Custom reference sets can be provided, and, going forward as more sequence data are generated and shared across the GPLN, this approach will be less limited in scope.

Piranha has been adopted for routine national sewage surveillance of Poliovirus in the UK, and staff from seventeen National Poliovirus Laboratories (NPLs) have been trained in the use of piranha, with implementation in six of these where it is being used to interpret data from routine stool surveillance or to validate implementation of DDNS. Staff trained were not bioinformaticians and would find command line coding challenging. PiranhaGUI facilitates far greater accessibility, allowing staff previously focused largely on cell culture and molecular laboratory methods to analyse large and complex sequencing datasets. Staff at the majority of NPLs would simply need to generate VP1 sequences for external transfer. A largely automated process (with the option for greater interaction by the command line) is therefore optimal over the employment of bioinformaticians to perform this repetitive task. Staff have been trained in quality control of the resulting sequencing data, with interpretation of positive and negative controls reflecting successful library preparation and the avoidance of contamination. Quality control of individual samples is also performed, with emphasis on biological interpretation, and potential contamination between samples is flagged and identified in the reports through highly similar sequences, which, for detections important to the GPLN (wild type and VDPVs), should vary between samples from different geographic regions. The phylogenetic tree produced by piranha facilitates this process.

Piranha can successfully identify poliovirus sequencing reads in samples for VP1 and whole genome sequencing of poliovirus and distinguish them from related non-polio enterovirus sequences at high specificity. It identifies mixtures of distinct poliovirus serotypes and strains within a sample, with potential signs of contamination flagged, and constructs consensus fasta sequences for each different poliovirus detected. Piranha presents the bioinformatic and phylogenetic analysis in an interpretable, actionable way that minimises the complexity of adopting a novel sequencing-based approach for poliovirus direct detection.

## Data Availability

Piranha is hosted on GitHub and available under a GNU General Public License v3.0. It is also hosted on Bioconda and has an associated Docker image hosted on Docker Hub.
